# Longitudinal rheumatoid factor autoantibody responses after SARS-CoV-2 vaccination or infection

**DOI:** 10.3389/fimmu.2024.1314507

**Published:** 2024-02-29

**Authors:** Sofie Keijzer, Nienke Oskam, Pleuni Ooijevaar-de Heer, Maurice Steenhuis, Jim B.D. Keijser, Luuk Wieske, Koos P.J. van Dam, Eileen W. Stalman, Laura Y.L. Kummer, Laura Boekel, Taco W. Kuijpers, Anja ten Brinke, S. Marieke van Ham, Filip Eftimov, Sander W. Tas, Gerrit J. Wolbink, Theo Rispens

**Affiliations:** ^1^ Department of Immunopathology, Sanquin Research and Landsteiner Laboratory, Academic Medical Center, Amsterdam, Netherlands; ^2^ Amsterdam Institute for Infection and Immunity, Amsterdam, Netherlands; ^3^ Department of Neurology and Neurophysiology, Amsterdam Neuroscience, Amsterdam UMC, Academic Medical Center, University of Amsterdam, Amsterdam, Netherlands; ^4^ Department of Rheumatology, Amsterdam Rheumatology and Immunology Center, Reade, Amsterdam, Netherlands; ^5^ Department of Pediatric Immunology, Rheumatology and Infectious Disease, Amsterdam UMC, Academic Medical Center, University of Amsterdam, Amsterdam, Netherlands; ^6^ Swammerdam Institute for Life Sciences, University of Amsterdam, Amsterdam, Netherlands; ^7^ Department of Rheumatology and Clinical Immunology, Amsterdam Rheumatology and Immunology Center, Amsterdam UMC, Academic Medical Center, University of Amsterdam, Amsterdam, Netherlands

**Keywords:** rheumatoid factor, vaccination, infection, autoantibodies, SARS-CoV-2, rheumatoid arthritis, autoimmunity

## Abstract

**Background:**

Rheumatoid factors (RFs) are autoantibodies that target the Fc region of IgG, and are found in patients with rheumatic diseases as well as in the healthy population. Many studies suggest that an immune trigger may (transiently) elicit RF responses. However, discrepancies between different studies make it difficult to determine if and to which degree RF reactivity can be triggered by vaccination or infection.

**Objective:**

We quantitatively explored longitudinal RF responses after SARS-CoV-2 vaccination and infection in a well-defined, large cohort using a dual ELISA method that differentiates between true RF reactivity and background IgM reactivity. In addition, we reviewed existing literature on RF responses after vaccination and infection.

**Methods:**

151 healthy participants and 30 RA patients were included to measure IgM-RF reactivity before and after SARS-CoV-2 vaccinations by ELISA. Additionally, IgM-RF responses after a SARS-CoV-2 breakthrough infection were studied in 51 healthy participants.

**Results:**

Published prevalence studies in subjects after infection report up to 85% IgM-RF seropositivity. However, seroconversion studies (both infection and vaccination) report much lower incidences of 2-33%, with a trend of lower percentages observed in larger studies. In the current study, SARS-CoV-2 vaccination triggered low-level IgM-RF responses in 5.5% (8/151) of cases, of which 1.5% (2/151) with a level above 10 AU/mL. Breakthrough infection was accompanied by development of an IgM-RF response in 2% (1/51) of cases.

**Conclusion:**

Our study indicates that *de novo* RF induction following vaccination or infection is an uncommon event, which does not lead to RF epitope spreading.

## 1 Introduction

Rheumatoid factors (RFs) are autoantibodies that target the Fragment crystallizable (Fc) region of IgG. RFs are associated with rheumatoid arthritis (RA), and are found in ~70% of RA patients. However, RFs are not specific for RA and are also found in other (autoimmune) conditions, such as Sjögren’s Syndrome (SS), systemic lupus erythematosus (SLE), and systemic sclerosis (SSc). Furthermore, RFs are found in the healthy population, where reported frequencies of RF-positive individuals range from 1 to 30% (1) and generally increase with age (2).

Despite their high prevalence, the function of RFs remains largely unknown. The association between increased RF levels and a poor disease prognosis in RA (3) indicates that RFs may play a role in the pathophysiology of RA. However, given that RFs are also observed in healthy people, they may be important in normal immunity as well. Here, RFs have been suggested to enhance immune complex formation and clearance, which could potentially help maintain immune homeostasis in reaction to, for instance, an infection (4, 5). In line with this hypothesis, many studies have reported an increased frequency of IgM-RF antibodies associated with an immune challenge such as an infection or vaccination. Regardless, reported RF prevalence or incidence varies greatly between studies, likely due to a combination of factors, including study design and assay technology. To obtain more insight in these associations, we reviewed the literature on this topic (see Results).

Due to the recent COVID-19 pandemic, the development of autoimmune responses after infection and vaccination has gathered renewed interest. Especially new-onset inflammatory arthritis is considered a relatively common consequence of COVID-19 (6, 7). RF frequencies in previously (seemingly) healthy individuals after SARS-CoV-2 infection are sometimes (7–10) – but not always – found to be increased (11–13). The discrepancies in existing literature make it difficult to determine to which degree vaccination or infection induce RF responses. Furthermore, current literature does not allow quantification or determination of RF reactivity and specificity, since information on the titer of these RF responses and development over time is often lacking.

We have recently developed a novel approach to map RF reactivity profiles, using engineered IgG target molecules that capture only specific subsets of RF specificities (14). In the process, we also created an ‘RF-dead’ IgG target, which was termed ‘IgG-Bare’, to which very little residual RF binding was observed. This target can conveniently be used as a negative control target, to distinguish between true RF reactivity and background, possibly polyreactive IgM reactivity.

In this study, we reviewed current literature on RF prevalence and seroconversion after infection and vaccination. Additionally, we determined longitudinal IgM-RF responses after SARS-CoV-2 vaccinations as well as after SARS-CoV-2 breakthrough infection, in a well-defined longitudinal cohort consisting of both healthy individuals and RA patients. We focus on the magnitude of the RF responses in addition to mere seroconversion, and make use of a dual ELISA approach to distinguish between true IgM-RF and background IgM reactivities over time.

## 2 Materials and methods

### 2.1 Literature search

PubMed was searched for relevant literature up to June 26, 2023. The keywords used for the search included ‘Rheumatoid Factor’, ‘Vaccine’, ‘Infection’, ‘SARS-CoV-2’, ‘Tetanus toxoid’, and ‘Hepatitis B’. We included studies which reported seroprevalence or seroconversion rates of RF after any infection or vaccination. Studies reporting RF seroconversion or seroprevalence only in the context of hepatitis C were excluded.

### 2.2 Study participants

Participants of this study are part of the ongoing Target-2-B! (T2B)! study, a large prospective multicenter cohort study in the Netherlands, focused on SARS-CoV-2 vaccination responses in patients with immune-mediated inflammatory disorders, as previously described (15, 16). In this substudy, we included 151 healthy controls from the T2B! cohort to explore RF responses after SARS-CoV-2 vaccination, and 51 healthy controls to study RF responses after SARS-CoV-2 breakthrough infection (i.e. a SARS-CoV-2 infection after completing primary SARS-CoV-2 vaccination). Healthy participants were excluded in case of active or previous autoimmune, oncological or hematological disease, or treatment with systemic immunosuppressive medication in the past year. Additionally, 30 patients with rheumatoid arthritis (RA) participating in the T2B! cohort were included. Participants received one or two of the following vaccines during the primary SARS-CoV-2 vaccine campaign in the Netherlands: BNT162b2 (Pfizer–BioNtech), CX-024414 (mRNA-1273; Moderna), ChAdOx1 nCoV-19 (Oxford–AstraZeneca) and Ad.26.COV2.S (Janssen, Johnson & Johnson). Second vaccination was optional for healthy individuals with a prior SARS-CoV-2 infection. An additional third (“booster”) vaccination was offered to all individuals in the Netherlands, and was administered at least three months after the last dose of SARS-CoV-2 vaccine. For the third vaccination, either BNT162b2 or CX-024414 was given. A SARS-CoV-2 breakthrough infection was confirmed by a positive PCR or antigen test at least 14 days after primary immunization occurring during follow-up time (between November 2021 and March 2022). All RA patients fulfilled the 2010 ACR-EULAR criteria and were under treatment with various types of systemic immunosuppressants. All participants of the study gave informed, written consent. The study was approved by the AMC medical ethical committee (NL74974.018.20 and EudraCT 2021-001102-30).

### 2.3 Sample collection

**Figure 1:**
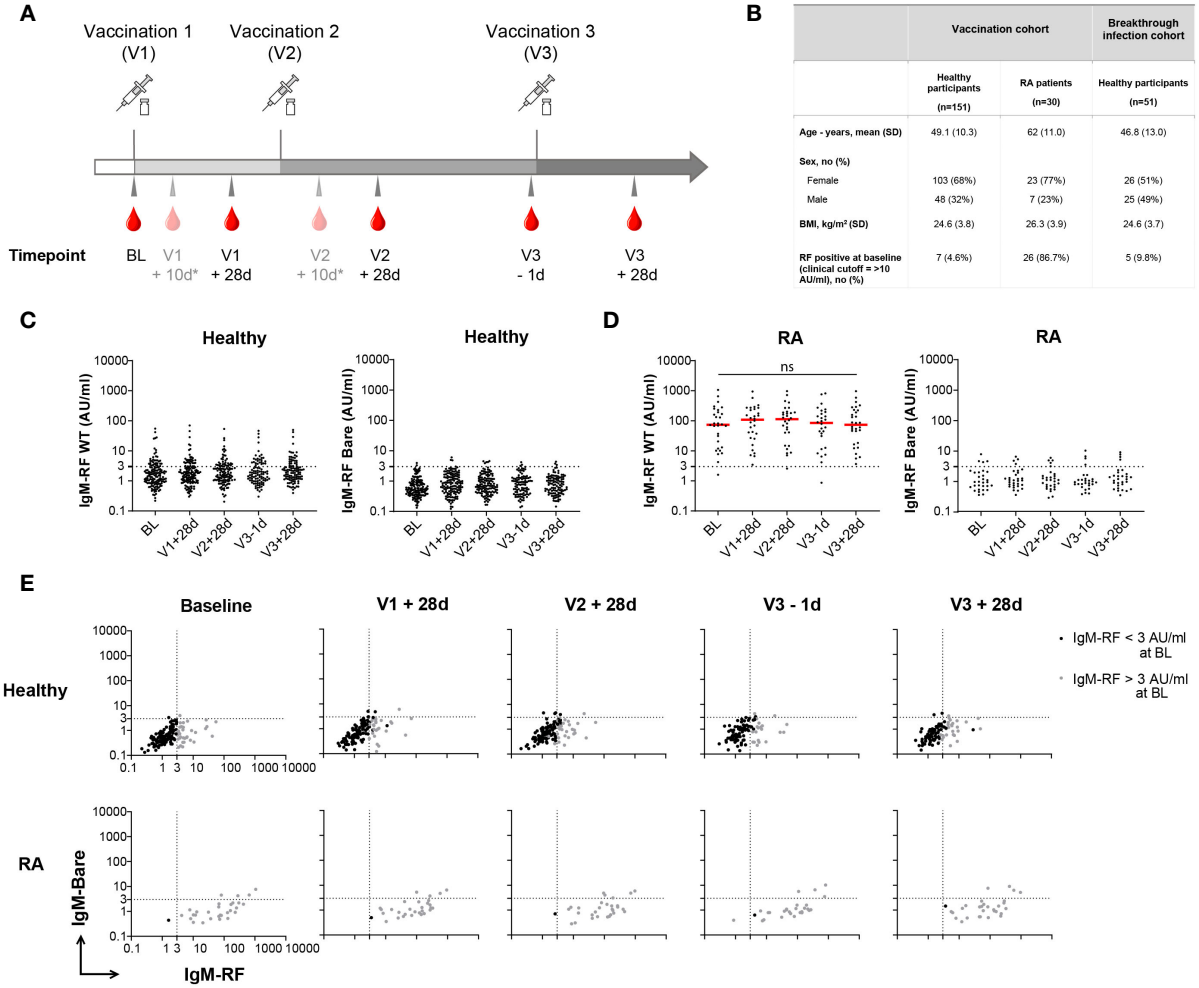
Longitudinal IgM-RF responses after SARS-CoV-2 vaccination. **(A)** Schematic overview of serum sample collection after vaccination. Timepoint V1 + 10d and V2 + 10d were collected for a subset of the healthy participant group. Median interval time for healthy participants between V1 and V2 is 42 days (IQR: 36-42), and between V2 and V3 190 days (IQR: 181-200). For RA patients median interval time between V1 and V2 is 37 days (IQR: 35-42), and between V2 and V3 185 days (IQR: 116-201). **(B)** Study participant baseline characteristics in the SARS-CoV-2 vaccination cohort and breakthrough infection cohort. RA patients were treated with immunosuppressive medication; 21/30 (70%) used methotrexate, 3/30 (10%) methotrexate + TNF inhibitors, and 6/30 (20%) other immunosuppressive medication. **(C)** IgM-RF levels (left panel) and IgM-RF levels against IgG-Bare (right panel) at different timepoints after vaccination in healthy participants. No difference in time points was observed based on Kruskal-Wallis test on the frequencies of positive samples per time point. **(D)** IgM-RF levels (left panel) and IgM-RF levels against IgG-Bare (right panel) at different timepoints after vaccination in RA patients. The median IgM-RF level for each timepoint is indicated in red. No significant differences in IgM-RF levels were observed between timepoints (Paired one-way ANOVA; F (2.108, 60.08) = 0.5783; P = 0.57) **(E)** IgM-RF reactivity against WT IgG and IgG-Bare, resembling the true and background target binding of IgM-RF, after vaccination in healthy participants and RA patients. Individuals with an IgM-RF level above the cutoff (>3 AU/mL) at baseline are indicated in grey to make tracking of individuals over time easier. ns, not significant.

Serum samples were collected from participants by venipuncture performed by a healthcare professional or were self-sampled at home by fingerprick (17). Serum samples were collected before SARS-CoV-2 vaccination (baseline), 10 and 28 days after first vaccination (V1 + 10d and V1 + 28d) and 10 and 28 days after second vaccination (V2 + 10d and V2 + 28d; if applicable; **Figure 1A**). For the third vaccination, serum samples were collected on the day of vaccination (V3 – 1d), and 28 days after the third vaccination (V3 + 28d). For participants of the breakthrough infection cohort, serum was collected at 0, 7, 28 and 90 days after a positive SARS-CoV-2 PCR or antigen test. Serum was collected and diluted 1:10 in phosphate buffered saline (PBS) supplemented with 0.1% Tween-20 and 2 g/L gelatin (PTG) and stored at -30°C.

### 2.4 Design of recombinant IgG targets

Recombinant RF target IgG antibodies were designed as previously described (14, 18). In addition to a wild type (WT) RF target IgG1 antibody, a “IgG-Bare” antibody was designed in which twenty human to murine IgG2b amino acid replacements in the Fc region resulted in low RF binding. Furthermore, RF target antibodies were designed in which several subsets of these mutations resulted in targets with locally restricted binding of RF within the Tail Region, Elbow Region, or Upper Region of the Fc region.

### 2.5 IgM-RF enzyme-linked immunosorbent assay

Serum IgM-RF binding to IgG was measured in an ELISA, as described previously (14, 18). The protocol was adjusted to half volume Corning Costar 96-wells ELISA plates (Thermo Fisher Scientific). In short, recombinant RF target IgG1 antibodies [produced as described in (14, 18)] were coated at 1 µg/mL in PBS overnight at 4°C. After washing with 0.02% Tween 20-PBS, 50 µl participant serum or a RF positive reference serum diluted in 0.1% Tween 20-PBS was incubated on the plates for 1h at RT while shaking. After washing, IgM-RF was detected by incubating with horseradish peroxidase (HRP)-conjugated mouse monoclonal anti-human IgM-Fc (0.33 µg/mL, MH-15-1; Sanquin) for 30 min at RT while shaking. As a coating control, coated IgG1 was detected with HRP-conjugated mouse monoclonal anti-human kappa (0.5 mg/mL, MH-19; Sanquin). The reaction was visualized with 50% 1-Step Ultra TMB-ELISA substrate (Sigma-Aldrich) diluted in MiliQ and stopped with 50 µL 0.2 M H_2_SO_4_. The optical density was read at 450 nm and 540 nm for background correction using a BioTek microtiter plate reader. Levels of IgM-RF were compared to a serial diluted reference serum (“Relares”) (19), containing an amount of IgM-RF that was set at 200 arbitrary units (AU) per mL.

### 2.6 Determination of IgM-RF assay cutoff

Within this study, IgM reactivity against WT IgG resembles specific target binding of RF, here referred to as ‘IgM-RF’ reactivity, while IgM reactivity against IgG-Bare represents background IgM-RF reactivity, in this paper referred to as ‘IgM-Bare’ reactivity. A strict, low cutoff for IgM-RF specificity of 3 AU/mL was defined based on IgM levels against IgG-Bare. This cutoff represents ca. 97% specificity, meaning ca. 97% of the individuals had reactivity against IgG-Bare below 3 AU/ml. The cutoff for specificity is used to distinguish between background IgM-RF responses and true IgM-RF responses.

Additionally, comparing our inhouse IgM-RF assay with clinical RF assays established that a cutoff of 10 AU/ml in our inhouse assay correlates with the cutoff for clinical RF positivity in clinical RF assays.

### 2.7 Statistical analysis

To compare the proportion of healthy individuals with positive IgM-RF levels (>3 AU/ml) at different timepoints to the proportion at baseline, a Kruskal-Wallis test with Dunn’s multiple comparisons test was performed on ordinal (positive or negative) transformed data. For RA patients a paired one-way ANOVA test was performed to analyze differences in IgM-RF levels at different timepoints after vaccination.

## 3 Results

### 3.1 Literature survey of association between infection/vaccination and RF

We reviewed literature on the association of RF induction by an immunological trigger, specifically infection and vaccination. The search of PubMed yielded a total of 35 relevant studies, of which 22 studies reported seroprevalence of RF, 10 studies reported seroconversion rates of RF and 3 studies reported RF+ B cells associated with vaccination or infection.

Many studies have described increased RF prevalence linked to infection. A specific case involves the well-studied, strong association of HCV infection with a distinct RF response (20, 21) that is highly restricted in its V gene usage (22), which will therefore not be further considered here. Multiple other types of both viral and bacterial infections, including HIV (23–25) and tuberculosis (26, 27), have been associated with a sometimes highly elevated prevalence of RF (for an overview see **Supplementary Table S1**), ranging from ~10% to up to 85% of cases. Several studies described higher RF titers in patients with various infections compared to non-infected individuals, for example in patients with COVID-19 (7–10), tuberculosis (26–28), endocarditis (29–31) and syphilis (32, 33). A number of studies have reported increased RF frequencies in previously healthy individuals after SARS-CoV-2 infection (7–10). Others, however, have reported similar RF levels among healthy controls and patients with COVID-19 (11–13).

In the context of vaccination, increased levels of RF precursor B cells were described after immunization of healthy participants with tetanus toxoid (TT) in particular (34, 35), albeit in small numbers of participants (**Supplementary Table S2**). Furthermore, multiple studies have been performed that specifically determined seroconversion following vaccination, and in a few cases, following an infection. An overview is provided in **Table 1**. Vaccines that have been studied include TT (37, 38), typhoid (39, 44) and influenza (40–42) vaccines. Only one study described seroconversion rates after SARS-CoV-2 vaccines (36). The presented rates of seroconversion vary between 2 and 33%. It should be pointed out however, that the higher numbers correspond to studies investigating relatively small groups of individuals of about 30 cases or less. Furthermore, in a few studies, seroconversion rates were investigated in a specific disease population such as RA or SLE, both systemic autoimmune diseases that are themselves already associated with a high prevalence of RF seropositivity. Taken together, although the emergence of novel RF responses is commonly reported after various infections and vaccinations, it is difficult to assess to what extent due to inconsistencies in assay setups, the lack of longitudinal sampling or the use of small cohorts.

**Table 1:** RF seroconversion studies associated with vaccination and infection.

Ref	Antigen/ pathogen	Study subjects	Vaccination/infection	Assay	Positive events	Findings
(12)	SARS-CoV-2	COVID-19 patients (n=33), pre-pandemic healthy controls (n=100)	Infection	ELISA	9% (3/33)	IgM-RF was detected in patients before and 7-11 months post infection. 3 out of 33 patients showed increases in RF, which did not significantly differ from pre-pandemic controls.
(13)	SARS-CoV-2	First-degree relatives of RA patients with (n=109) and without SARS-CoV-2 infection (n=59)	Infection	ELISA	2.8% (3/109) with SARS-CoV-2 infection, 8.5% (5/59) without infection.	IgM-RF positivity did not significantly change after infection. No information on titers.
(36)	BNT162b2 mRNA COVID-19 vaccine	Autoimmune inflammatory rheumatic disease patients (n=463)	Vaccination	Nephelometry	0.4% (2/461) after two doses 3.4% (12/463) after three doses	3.4% of patients were newly seropositive for RF after three doses of vaccine. No information on titers.
(37)	Tetanus toxoid, *S. typhi*, Mumps (Enders strain), Diphtheria toxoid, Polio virus, smallpox	Healthy subjects (n=245)	Vaccination	Waaler-Rose and Latex agglutination	3% (8/245)	Transient increases in RF were observed in 8 subjects. Titers given, variable.
(38)	Tetanus toxoid, *S. typhi*, Mumps (Enders strain), Diphtheria toxoid, Polio virus, smallpox	Healthy subjects (n=381)	Vaccination	Waaler-Rose and Latex agglutination	2% (8/381)	Transient increases in RF were observed in 8 subjects in total. Titers not given.
(39)	Typhoid vaccination	Healthy adults receiving oral (n=30) or parenteral (n=30) vaccine	Vaccination	ELISA	Unknown	Oral group: no increase in median RF titer Parenteral group: relative increase in median titer ca. 2-fold
(40)	Influenza split virion inactivated vaccine	RA patients (n=82)	Vaccination	ELISA	Unknown	Mean RF levels were increased upon vaccination, but this was not significant.
(41)	Influenza H1N1 vaccine	SLE patients (n=89)	Vaccination	Nephelometry	12% (11/89)	RF positivity pre-vaccination was 15,7% (14/89), and post-vaccination 28.1% (25/89). Mean RF level remained unchanged between visits.
(42)	Influenza A2 Virus	Influenza experienced individuals (n=27), healthy controls (n=79)	Infection/Vaccination	Waaler-Rose and Latex agglutination	14.8% (4/27) of influenza experienced subjects, 1.3% (1/79) of healthy subjects	RF levels were higher in Influenza experienced participants (2 participants vaccinated, 2 participants infected) compared to non-infected/vaccinated controls. Titers given, fairly low.
(43)	Brucella vaccine	RA patients (n=103), healthy relatives of RA patients (n=91), healthy subjects (n=106)	Vaccination	Waaler-Rose and Latex agglutination	Waaler-Rose: 33% (7/21) of initially seronegative RA patients, 16% (14/91) of relatives, 3% (3/106) of healthy subjects. Latex: 2/103; 2/91; 1;106, i.e., 1-2%	After an injection of brucella vaccine RF levels increased in all groups. Waaler-Rose higher seroconversion rates than Latex agglutination test. Only mean titers are given, modest increase (<2-fold)

### 3.2 IgM-RF responses following SARS-CoV-2 vaccination

In order to gain better insight into these possible *de novo* RF responses, we investigated longitudinal RF responses after vaccination in a study cohort consisting of 151 healthy individuals (15) and 30 RA patients. Baseline characteristics of the participants are shown in **Figure 1B**. **Figure 1A** shows the timeline for collection of serum samples from participants, comprising baseline (BL), 28 days after the first and second vaccination (V1 + 28d and V2 + 28d), before the third vaccination (V3 – 1d), and 28 days after the third SARS-CoV-2 vaccination (V3 + 28d).

IgM-RF was assessed as IgM binding to human recombinant IgG1 by ELISA. As a negative control, we also measured IgM binding to our previously described ‘IgG-Bare’ target (14). RF binding to IgG-Bare is almost completely abolished due to replacement of twenty human amino acid residues with their mouse IgG2b analogs. At baseline, IgM-RF levels in healthy participants were low, as expected, with <5% of participants exceeding levels of 10 AU/mL, which is in this assay the RF level correlating with the cutoff for clinical RF positivity. Furthermore, 23% of healthy participants showed IgM-RF levels >3 AU/ml. This percentage did not significantly change at following timepoints (**Figure 1C**). IgM-RF reactivity against IgG-Bare was also low and markedly decreased compared to reactivity against WT IgG (**Figure 1C**; **Supplementary Figure S1A**). At all timepoints after vaccination, IgM-RF levels were similar to baseline (**Figure 1C**; **Supplementary Figure S1B**). This indicates that overall, no substantial *de novo* IgM-RF response is induced upon vaccination. In RA patients, RF levels were much higher compared with healthy individuals, i.e. 97% showed IgM-RF levels >3 AU/ml, but did not change significantly after repeated vaccinations compared to baseline (**Figure 1D**; **Supplementary Figures S1A**, **S1C**).

In order to better visualize the trend of RF responses after vaccination over time for each individual, IgM reactivity against WT IgG and IgG-Bare, resembling the true IgM-RF target binding and background IgM-RF binding, were compared for each healthy participant and RA patient (**Figure 1E**). Within this study, a strict, low cutoff for IgM-RF specificity of 3 AU/mL was defined based on IgM levels against IgG-Bare. This cutoff represents ca. 97% specificity, meaning ca. 97% of the individuals had background IgM reactivity against IgG-Bare below 3 AU/ml. 23% of the healthy participants had IgM-RF levels above the cutoff at baseline (>3 AU/mL), and 5% showed IgM-RF levels above 10 AU/ml. We observed that healthy participants with IgM-RF levels above 3 AU/mL consistently remained above 3 AU/mL within the duration of the study (**Supplementary Figure S1D**). Development of *de novo* RF reactivity against WT IgG after vaccination was only observed at low levels, between 3 and 10 AU/mL, except for two individuals. Furthermore, part of this very modest increase in reactivity coincided with elevated background IgM reactivity against the IgG-Bare target, indicating that even this small increase in reactivity only partly represents true RF reactivity.

**Figure 2:**
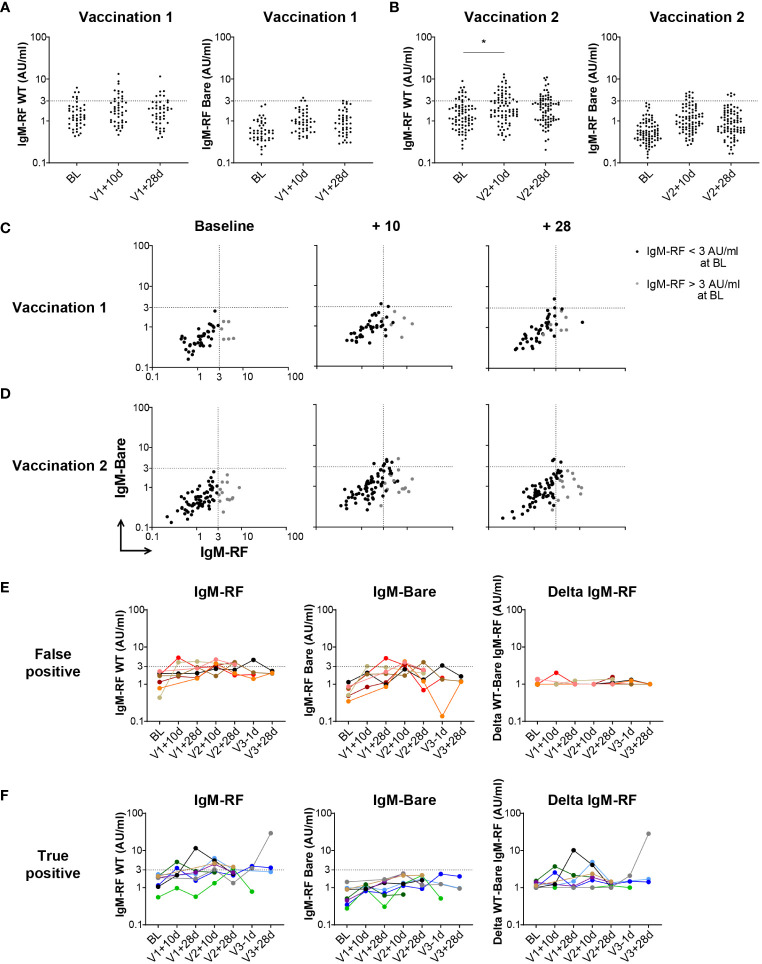
Early IgM-RF responses after SARS-CoV-2 vaccination. **(A)** IgM-RF levels (left panel) and IgM-RF levels against IgG-Bare (right panel) at 10 days and 28 days after the first SARS-CoV-2 vaccination in a subset of healthy participants (n=45). **(B)** IgM-RF levels (left panel) and IgM-RF levels against IgG-Bare (right panel) at 10 days and 28 days after the second SARS-CoV-2 vaccination in a subset of healthy participants (n=80). Frequencies of positive samples per time point were analyzed using Kruskal-Wallis test, * < 0.05. **(C, D)** IgM-RF reactivity against WT IgG and IgG-Bare, resembling the true and background target binding of IgM-RF, 10 days and 28 days after first vaccination (panel **C**) and second vaccination (panel **D**) in healthy participants. Individuals with an IgM-RF level above the cutoff (>3 AU/mL) at baseline are indicated in grey to make tracking of individuals over time easier. **(E, F)** Cases of vaccine-induced IgM-RF responses. We selected individuals in which WT IgM-RF levels increased >2-fold relative to baseline, thereby reaching levels above 3 AU/mL, at any time point after vaccination. Of these 15 cases, seven also developed background IgM-Bare reactivity, suggesting the increase in IgM-RF reactivity does not represent true RF (‘false positive’; panel **E**), whereas eight cases developed only WT IgM-RF reactivity, meaning a true positive increase in IgM-RF reactivity (panel **F**). IgM-RF levels (left panel), IgM-RF levels against IgG-Bare (middle panel) and delta of IgM-RF minus background IgM-Bare levels (right panel) at different timepoints are shown. For the delta IgM-RF levels, extremely low and negative delta levels are set at 1 AU/ml.

Although we did not identify a substantial increase in IgM-RF after vaccination, a complicating factor may be the relatively late sampling time of 28 days after SARS-CoV-2 vaccination at which point a very short-lived, transient IgM-RF response may have disappeared again (37). Therefore, IgM-RF levels were also measured at two additional, earlier timepoints for the subsets of the healthy participants for which such an additional sampling time point was available, namely at 10 days after the first or second vaccination, in addition to the 28 days post vaccination timepoints. The proportion of positive IgM-RF levels after both the first and second SARS-CoV-2 vaccination were increased to 27% and 34% at V1 + 10d and V2 + 10d, respectively, compared to 23% at baseline, which was significant only at V2 + 10d (p<0.05; **Figures 2A, B**; **Supplementary Figure S2**; **Supplementary Table S3**). This transient increase in IgM-RF reactivity against WT IgG remained below 10 AU/ml and coincided in part with increased reactivity against IgG-Bare (**Figures 2C, D**). Thus, an early increase in IgM-RF levels in healthy individuals after vaccination was only observed to a minor degree, and in all cases with levels below 10 AU/mL.

To better examine potential cases of (transient) vaccine-induced RF responses, we selected individuals in which WT IgM-RF levels increased >2-fold relative to baseline, and thereby reached levels above 3 AU/mL at any time point after vaccination. Of these 15 cases (10%) in which WT IgM-RF levels increased, seven (4.5%) also developed reactivity against IgG-Bare, suggesting the increase in IgM-RF reactivity does not represent true RF (‘false positive’; **Figure 2E**). On the other hand, eight (5.5%) cases only developed IgM-RF reactivity against WT IgG, representing true positive increases in IgM-RF reactivity (‘true positive’; **Figure 2F**). The number of individuals with a positive increase in IgM-RF reactivity compared to baseline is summarized for each timepoint in **Supplementary Table S3**. There was no overall consistent pattern, and responses were invariably detectable only at some, but not all, timepoints after vaccination.

### 3.3 IgM-RF responses following SARS-CoV-2 infection

**Figure 3:**
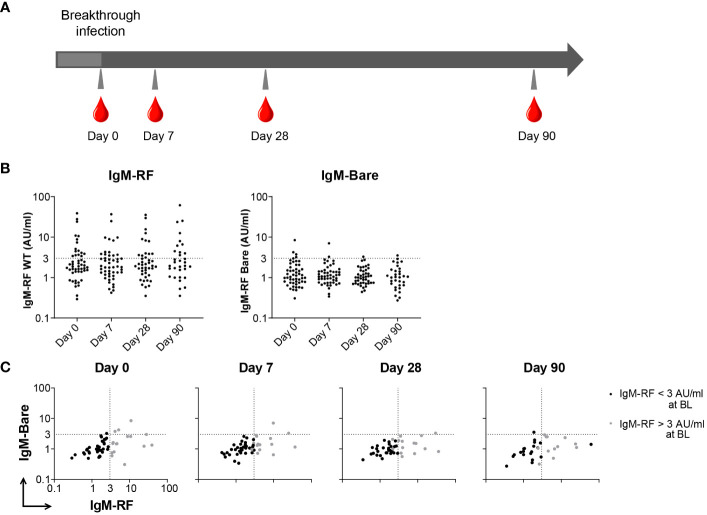
IgM-RF responses after SARS-CoV-2 breakthrough infection. **(A)** Schematic overview of serum sample collection after breakthrough infection. **(B)** IgM-RF levels (left panel) and IgM-RF levels against IgG-Bare (right panel) at different timepoints after infection in healthy participants. **(C)** IgM-RF and IgM-Bare reactivity after breakthrough infection. Individuals with an IgM-RF level above the cutoff (>3 AU/mL) at baseline are indicated in grey to make tracking of individuals over time easier.

In addition to vaccination, we also investigated whether a SARS-CoV-2 infection induced IgM-RF responses. In order to study RF levels after infection, longitudinal samples of 51 healthy controls before and after a SARS-CoV-2 breakthrough infection were analyzed (16). Baseline characteristics for these participants can be found in **Figure 1B**. The majority of breakthrough infections occurred between January and March 2022, when the BA.1 variant was predominant in The Netherlands. IgM-RF reactivity was measured at different timepoints after a breakthrough infection up to 90 days after a positive SARS-CoV-2 test (**Figure 3A**). 31% of the healthy participants showed IgM-RF levels above 3 AU/ml at the day of their positive SARS-CoV-2 test (**Figure 3B**; **Supplementary Table S3**). In line with the results after vaccination, the proportion of individuals with measurable serum IgM-RF levels remained almost constant after a breakthrough infection (**Supplementary Table S3**). Only one case of IgM-RF induction was observed. Overall, SARS-CoV-2 infection did not induce IgM-RF responses in the vast majority of healthy participants (**Figures 3B, C**; **Supplementary Figures S3A, B**).

### 3.4 IgM-RF binding patterns on the IgG-Fc after SARS-CoV-2 vaccination

We have previously mapped the polyclonal RF repertoires in sera from RA patients and healthy donors and observed that RFs from these groups targeted distinct epitopes within the IgG-Fc (14, 18). Interestingly, some healthy participants from the T2B! cohort study already showed IgM-RF levels at baseline above the defined cutoff. We determined the RF binding patterns for these individuals and a subset of RA patients at baseline and post second or third vaccination. In line with our previous study (14), RF from these healthy participants target mainly binding epitopes within the lower IgG-Fc, while those from RA patients bind slightly higher (**Supplementary Figure S4**). Moreover, differences between RF binding epitopes before and after vaccination were not observed in healthy participants nor RA patients, which implies that epitope spreading is not induced to any significant degree following vaccination in individuals that were already RF positive.

## 4 Discussion

Multiple studies have reported on the emergence of (transient) RF responses after infection or vaccination (**Table 1**). Recently, these findings have been extended to SARS-CoV-2, were the presence of RF is strongly implied, albeit at different rates and without information on the magnitude of the RF responses (7–10). We therefore investigated longitudinal IgM-RF responses in a well-defined, large cohort of healthy subjects and RA patients after SARS-CoV-2 vaccination or breakthrough infection. Our data show that IgM-RF responses were only induced in a small subset, mostly transiently and at very low levels, and lacking a consistent pattern over time. Whether these increases are relevant, and possibly pathologic, is difficult to assess as clinical thresholds vary per RF test and are often insufficiently defined due to poor test standardization (45). However, most of the newly emerging reactivity remained below 10 AU/mL (ca. 95% of healthy individuals scored below 10 AU/mL at baseline). These data extend previous studies. In summary, they indicate that the emergence of a (transient) RF response is relatively uncommon and does not consistently accompany an adaptive immune response, as has been previously suggested by others (1).

In the present study, we used a dual ELISA approach to distinguish between well-established, true RF binding and background IgM binding. We observed that the low level of IgM-RF reactivity to IgG that emerges following an immune trigger partly represents background IgM binding. These results indicate that rather than specific autoantibody induction, an immune challenge may instead be accompanied by temporarily elevated, ‘polyreactive’ IgM production, or a certain level of bystander B cell activation. The short-lived, transient IgM-RF responses after vaccination could possibly serve to moderate immune homeostasis by helping with the clearance of immune complexes, but the function of these RFs remains debated. Nevertheless, RFs are sometimes also classified as polyreactive, and the distinction is probably not absolute.

Previous studies that showed substantially increased RF levels in patients with/post COVID-19 as compared to healthy controls (7–10), are in line with similar studies performed in the context of other infections (**Supplementary Table S1**). However, with respect to the reviewed studies in this paper, there are several points to consider. As mentioned earlier, RF prevalence studies lack baseline measurements and therefore cannot distinguish between RF that was already present in patients and *de novo* RF responses. It is for instance not unlikely that patients with underlying (and undiagnosed) autoimmune disorders are more susceptible to SARS-CoV-2 infections and may therefore be overrepresented in the patient group. Furthermore, while seroconversion rates are reported in a number of these studies (**Table 1**), information on the titer of these responses and development over time is often lacking. Therefore, it is often unclear if the RF responses following vaccination or infection are very weak, transient reactivities or represent a substantial, lasting RF response. Moreover, the techniques used to measure RF vary, and include sheep red blood cell agglutination (Waaler-Rose test), latex agglutination, nephelometry and ELISA assays. Notably, one study using both the Waaler-Rose test and the latex agglutination test observed substantial discrepancies in incidence (43). This indicates that specificity of the assays may vary substantially. Given this lack of assay consistency, our inhouse IgM-RF assay was previously compared to four commercially available RF tests (45). Although results showed that the RF target antigen used was an important cause of some discrepancies, the assays are largely comparable, especially those using human IgG as RF target.

A concern with the emergence of SARS-CoV-2 and related mRNA vaccines is the possible development of autoimmune disorders, either as a consequence of molecular mimicry or overactivation of the immune system. Especially the new-onset of (inflammatory) arthritis has been reported frequently, but inconsistently (6). Our results suggest that true *de novo* induction of RF reactivity is probably a rare event. Furthermore, when we determined RFs targeting an RA-specific epitope (14) in healthy participants with detectable RF levels, we found that this reactivity was not induced by either infection or inflammation. This suggests that in individuals already positive for RF, epitope spreading is not typically induced by infection or vaccination. This is in line with studies showing little seroconversion for ACPA as well and very few patients actually developing arthritis after SARS-CoV-2 infection or vaccination (13, 46).

Serum IgM-RF levels increase with age (2), therefore, we examined if the age of our participants might have an impact on positive IgM-RF reactivity. However, we did not find an increase in IgM-RF levels with increasing age of the healthy participants in the vaccination cohort.

Although other isotypes of RF have been described, in this study, exclusively RF of the IgM isotype was investigated. A previous study has reported that the IgM-RF repertoire binds to a broader range of epitopes than the IgA-RF repertoire, which is more restricted (14). Furthermore, very low IgA-RF reactivity was observed in healthy subjects, suggesting high IgA-RF levels might be linked to pathogenesis. Therefore, we expect it to be difficult to measure distinct IgA-RF dynamic changes after vaccination or infection in healthy individuals. However, since SARS-CoV-2 primarily affects mucosal surfaces, this might have an effect on IgA-RF, but this has to be examined further. IgG-RF measurements are technically very difficult, and assays are poorly standardized, therefore, RF of the IgG isotype was also not studied.

Overall, careful examination in a prospective cohort including participants after SARS-CoV-2 vaccination and breakthrough infection shows no evidence for the consistent emergence of *de novo* RF autoreactivity nor RF epitope spreading.

## Data availability statement

The raw data supporting the conclusions of this article will be made available by the authors upon request.

## Ethics statement

The studies involving humans were approved by Amsterdam medical center medical ethical committee. The studies were conducted in accordance with the local legislation and institutional requirements. The participants provided their written informed consent to participate in this study. No animal studies are presented in this manuscript.

## Author contributions

SK: Conceptualization, Data curation, Investigation, Writing – original draft, Writing – review & editing. NO: Conceptualization, Writing – original draft, Writing – review & editing. PH: Investigation, Writing – review & editing. MS: Conceptualization, Writing – review & editing. JK: Writing – review & editing. LW: Resources, Writing – review & editing. KD: Data curation, Writing – review & editing. ES: Data curation, Writing – review & editing. LK: Data curation, Writing – review & editing. LB: Data curation, Writing – review & editing. TK: Writing – review & editing. AB: Writing – review & editing. SH: Writing – review & editing. FE: Writing – review & editing. ST: Writing – review & editing. GW: Writing – review & editing. TR: Conceptualization, Methodology, Supervision, Writing – original draft, Writing – review & editing.

## T2B! Immunity against SARS-CoV-2 study group

Anneke J. van der Kooi, Joop Raaphorst, Mark Löwenberg, R. Bart Takkenberg, Geert R.A.M. D’Haens, Phyllis I. Spuls, Marcel W. Bekkenk, Annelie H. Musters, Nicoline F. Post, Angela L. Bosma, Marc L. Hilhorst, Yosta Vegting, Frederike J. Bemelman, Alexandre E. Voskuyl, Bo Broens, Agner R. Parra Sanchez, Cécile A.C.M. van Els, Jelle de Wit, Abraham Rutgers, Karina de Leeuw, Barbara Horváth, Jan J.G.M. Verschuuren, Annabel M. Ruiter, Lotte van Ouwerkerk, Diane van der Woude, Renée C.F. van Allaart, Y.K. Onno Teng, Pieter van Paassen, Matthias H. Busch, Papay B.P. Jallah, Esther Brusse, Pieter A. van Doorn, Adája E. Baars, Dirk Jan Hijnen, Corine R.G. Schreurs, W. Ludo van der Pol, H. Stephan Goedee, Koos A.H. Zwinderman, Zoé L.E. van Kempen, Joep Killestein, Adriaan G. Volkers, Laura Fernandez Blanco, Niels J.M. Verstegen, Olvi Cristianawati.
