# Supplementary material for: Longitudinal rheumatoid factor autoantibody responses after SARS-CoV-2 vaccination or infection
Source: Front Immunol. 2024 Feb 29;15:1314507. doi: 10.3389/fimmu.2024.1314507 (PMC10937420; doi:10.3389/fimmu.2024.1314507)
Supplement: Supplementary file 1 [file DataSheet_1.docx]

Supplementary Material

# Supplementary Tables

## Supplementary Table 1. Seroprevalence studies of RF associated with infection. HCV not included.

| Ref | Antigen/  pathogen | Study subjects | Assay | Positive events | Findings |
| --- | --- | --- | --- | --- | --- |
| (7) | SARS-CoV-2 | Post-COVID-19 patients (n=100) | Nephelometry | 19% (19/100) | Patients that had prior COVID-19 and developed arthritis had higher frequencies of RF. |
| (8) | SARS-CoV-2 | COVID-19 patients (n=129 of which 5 were monitored longitudinally) | ELISA | 20.16% (26/129) | In 20.16% of COVID-19 patients RF was detected. In five longitudinally monitored patients, RF was found to be present not only in the acute phase of COVID, but also during the time of recovery. |
| (9) | SARS-CoV-2 | Convalescent COVID-19 patients (n=201),  healthy subjects (n=36) | ELISA | 20% of COVID-19 patients,  0% of healthy controls | The frequency of RF was increased in participants with prior SARS-CoV-2 compared to healthy controls. |
| (10) | SARS-CoV-2 | COVID-19 patients (n=120), pre-pandemic healthy controls (n=100) | ELISA | 25.8% (31/120) of COVID patients, 14% (14/100) of healthy subjects | The frequency of RF was increased in COVID-19 patients compared to pre-pandemic controls. |
| (11) | SARS-CoV-2 | Convalescent mild COVID-19 patients (n=68),  acute severe COVID-19 patients (n=12),  healthy subjects (n=39) | ELISA | Unknown | Levels of RF were not significantly different in COVID-19 (mild or severe) and healthy subjects. |
| (47) | Various viruses | Rubella (n=43), influenza A or B (n=33), cytomegalo- (n=17), herpes simplex (n=17), respiratory syncytial (n=16), parainfluenza (n=13), adeno- (n=10) and mumps (n=10) virus infected patients | ELISA | Unknown | Levels of IgM-RF were significantly increased in patients infected with Rubella and Influenza. |
| (48) | HBV and HCV | HBV patients (n=44),  HCV patients (n=43),  RA patients (n=25),  healthy subjects (n=46) | Nephelometry | 11.4% (5/44) of HBV patients,  16.3% (7/43) of HCV patients,  60% (15/25) of RA patients,  2.2% (1/46) of healthy controls | RF prevalence was higher in HBV and HCV patients compared to healthy controls. |
| (49) | HIV, HBV, HCV | HIV patients (n=31),  HBV patients (n=180),  HCV patients (n=18),  healthy subjects (n=176) | Latex agglutination | 9.8% (3/31) of HIV patients,  8.9% (16/180) of HBV patients, 27.7% (5/18) of HCV patients | IgM RF levels were not significantly different in patients infected with HIV or HBV compared to healthy volunteers. However, infection by HCV induced the production of IgM RF in the study subjects. |
| (23) | HIV | HIV infected children (n=24), uninfected children from HIV-positive mothers (n=22) | ELISA | 50% (12/24) of HIV patients,  0% of controls | HIV-infected children were mainly positive for IgA-RF. |
| (24) | HIV | AIDS patients (n=16), patients with AIDS-related complex (ARC) (n=32), healthy subjects (n=40) | ELISA | 68.7% (11/16) of AIDS patients,  62.5% (20/32) of ARC patients | IgM-RF and IgA-RF were increased in both AIDS and ARC patients compared to healthy controls, with no major differences between AIDS and ARC. |
| (25) | HIV | AIDS patients (n=34),  healthy subjects (n=43) | RIA & ELISA | 26% (9/34) of AIDS patients,  0% of healthy controls for IgA-RF | Only IgA-RF was increased in patients with AIDS compared to healthy controls. IgM-RF levels were low did not differ between groups. |
| (26) | *Mycobacterium tuberculosis* | TB patients (n=47), healthy subjects (n=39) | ELISA | 62% (29/47) of TB patients,  2.6% (1/39) of controls | IgM-RF prevalence was higher in TB patients compared to healthy individuals. |
| (27) | *Mycobacterium tuberculosis* | TB patients (n=19),  healthy subjects (n=25) | Unknown | 21% (4/19) of TB, 0/25 of healthy subjects | Mean levels of RF were significantly higher in TB patients than in healthy controls. |
| (28) | *Mycobacterium tuberculosis* | TB infected patients (n=3), RA patients (n=4),  healthy subjects (n=4) | ELISA | Unknown | RF production was higher in B cells obtained from TB and RA patients compared to healthy controls. |
| (29) | *Staphylococcus aureus* endocarditis | Drug abusers with endocarditis (n=55),  drug abusers without endocarditis (n=30) | Latex agglutination | 24% (13/55) of endocarditis patients, 7% (2/30) of noninfected people | RF positivity was higher in endocarditis patients compared to noninfected people. |
| (30) | Bacterial endocarditis | Subacute bacterial endocarditis (n=119),  drug abusers without endocarditis (n=11),  healthy subjects (n=13) | RIA | 72% (18/25) | IgM- and IgG-RF positivity was higher in endocarditis patients compared to noninfected people. |
| (50) | Bacterial endocarditis | Endocarditis patients (n=51) | Latex agglutination | 50% (26/50) | In half of the endocarditis patients RF was observed. |
| (51) | Varia | Endocarditis patients (n=22) | Latex agglutination | 45.5% (10/22) of endocarditis patients | In approx. half of the endocarditis patients RF was observed. |
| (31) | Varia | Endocarditis patients (n=56), rejected endocarditis patients (n=214) | Unknown | 36% (21/56) of endocarditis patients,  19% (36/214) rejected patients | RF was increased in patients with diagnosed endocarditis compared to patients that did not receive the diagnosis |
| (32) | *Treponema pallidum* | Syphilis patients (n=53),  RA patients (n=39),  healthy subjects (n=10) | Latex agglutination | 26.4% (14/53) of syphilis patients, 41% (16/39) of RA patients,  0% (0/10) of healthy controls | Increased RF levels were observed in syphilis patients compared to healthy controls. |
| (33) | *Treponema pallidum* | Infants with congenital syphilis (n=41),  healthy subjects (n=176) | ELISA | 35/41 (85%) | IgM-RF was detectable in 35/41 patients at levels above 95 percentile of healthy subjects |
| (52) | *Loa loa* | Loiasis patients (n=45) | ELISA | Unknown | Mean levels of IgM-RF and IgG-RF were higher in patients with Loiasis than healthy controls. |

## Supplementary Table 2. Studies reporting RF+ B cells associated with vaccination.

| Ref | Antigen/  pathogen | Study subjects | Vaccination/infection | Assay | Positive events | Baseline (yes/no) | Findings |
| --- | --- | --- | --- | --- | --- | --- | --- |
| (34) | Tetanus toxoid | Healthy adults (n=6) | Vaccination | ***Analysis of RF-secreting cells*** | Unknown | Yes | The number of RF-secreting cells transiently increased after a tetanus toxoid booster immunization. However, none of the individuals studied showed increased RF levels in serum. |
| (35) | Tetanus toxoid | Healthy adults (n=8) | Vaccination | ***Analysis of RF-secreting cells*** | Unknown | Yes | The frequency of IgM RF precursor B cells increased in tetanus toxoid vaccinated healthy adults up to 18 days after vaccination, which was shown after *in vitro* stimulation of the cells with EBV. In contrast, plasma IgM-RF levels increased only transiently. |
| (53) | Tetanus toxoid | Healthy adults (n=36) | Vaccination | ***Analysis of RF-secreting cells*** | 27.8% (10/36) | No | *In vitro* tetanus toxoid booster stimulation of PBMCs induced IgM-RF production in 10/36 experiments. |

## Supplementary Table 3. Number of healthy participants in the vaccination cohort and breakthrough infection cohort with positive IgM-RF levels for each timepoint.

Total number of individuals with positive IgM-RF levels (>3 AU/ml) are indicated for all timepoints, as well as the percentage of positive cases. Furthermore, the number of vaccination or infection induced IgM-RF responses is indicated for each timepoint. We selected individuals in which WT IgM-RF levels increased >2-fold relative to baseline, thereby reaching levels above 3 AU/mL, at any time point after vaccination. In case of a ‘false positive’ IgM-RF response background IgM-Bare reactivity also developed at that timepoint, while for a ‘true positive’ only WT IgM-RF reactivity developed.

|  | **Vaccination cohort** | | | | | | | **Breakthrough infection cohort** | | | |
| --- | --- | --- | --- | --- | --- | --- | --- | --- | --- | --- | --- |
|  | BL | V1 + 10d | V1 + 28d | V2 + 10d | V2 + 28d | V3 - 1d | V3 + 28d | Day 0 | Day 7 | Day 28 | Day 90 |
| *Healthy* |  |  |  |  |  |  |  |  |  |  |  |
| n= | 151 | 48 | 139 | 89 | 129 | 98 | 107 | 51 | 49 | 43 | 32 |
| no. positive  (>3 AU/ml) | 35 | 12 | 35 | 27 | 37 | 27 | 29 | 16 | 14 | 13 | 11 |
| % positive | 23% | 27% | 25% | 34% | 29% | 28% | 27% | 31% | 29% | 30% | 34% |
| no. positive increase relative to baseline | - | 4 | 2 | 7 | 4 | 2 | 2 | - | 0 | 0 | 1 |
| no. true positive | - | 2 | 1 | 4 | 1 | 1 | 2 | - | 0 | 0 | 1 |
| no. false positive | - | 2 | 1 | 3 | 3 | 1 | 0 | - | 0 | 0 | 0 |

# Supplementary Figures


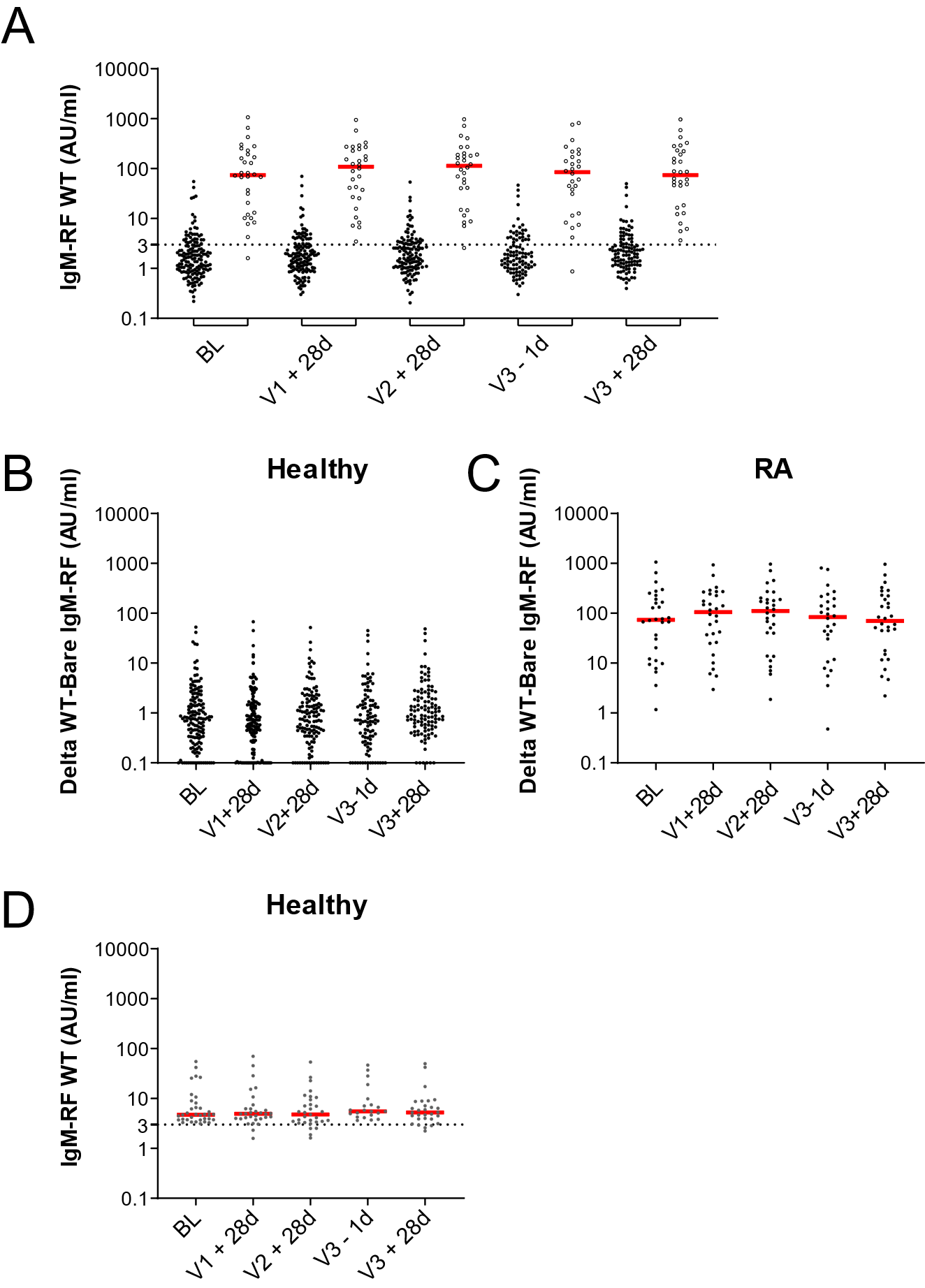


## Supplementary Figure 1.

**(A)** IgM-RF levels at different timepoints after vaccination in healthy participants (in black) and RA patients (in white). The median IgM-RF level for each timepoint is indicated in red. **(B)** Delta of the IgM-RF levels minus IgM-RF levels against IgG-Bare at different timepoints after vaccination for healthy participants. Extremely low and negative delta levels below 0.1 AU/ml are set at 0.1 AU/ml. **(C)** Delta of the IgM-RF levels minus IgM-RF levels against IgG-Bare at different timepoints after vaccination for RA patients. **(D)** IgM-RF levels at different timepoints of exclusively the healthy participants who have an IgM-RF level above the cutoff (>3 AU/mL) already at baseline. The median IgM-RF level for each timepoint is indicated in red.


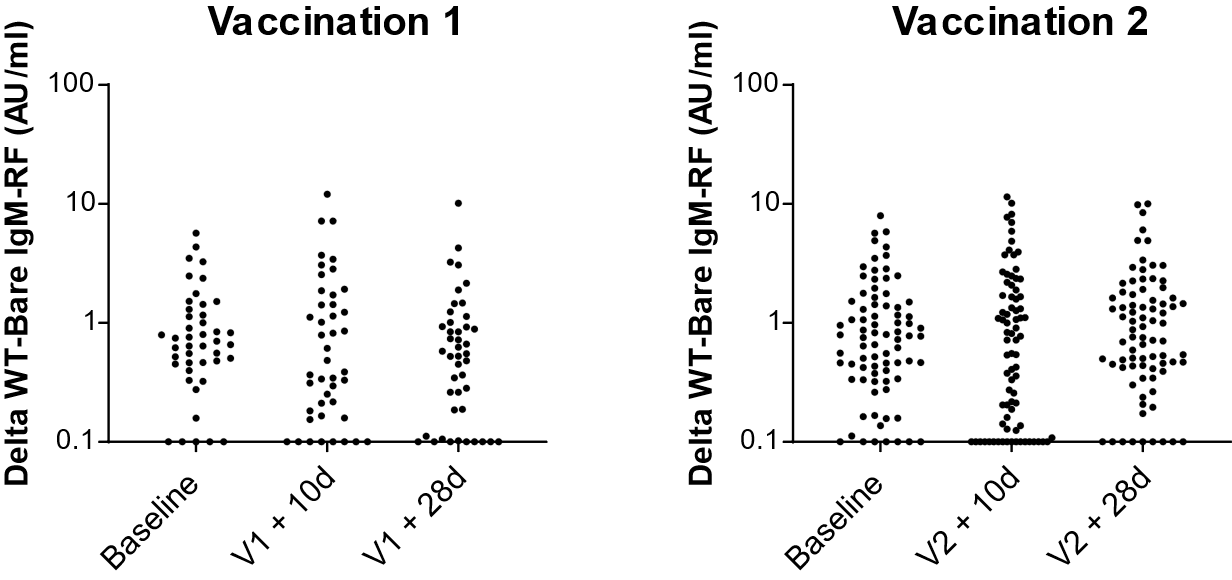


## Supplementary Figure 2.

Delta of the IgM-RF levels minus IgM-Bare levels at 10 days and 28 days after the first (left panel) and second (right panel) SARS-CoV-2 vaccination in a subset of healthy participants. Extremely low and negative delta levels below 0.1 AU/ml are set at 0.1 AU/ml.


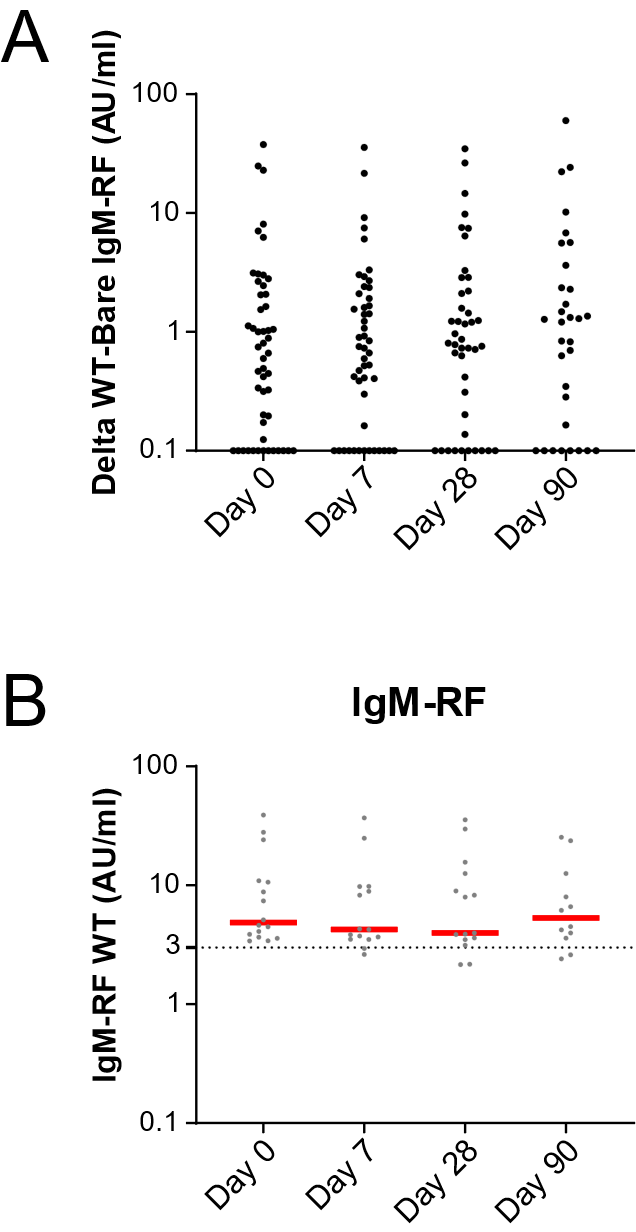


## Supplementary Figure 3.

**(A)** Delta of the IgM-RF levels minus IgM-Bare levels at different timepoints after breakthrough infection for healthy participants. Extremely low and negative delta levels below 0.1 AU/ml are set at 0.1 AU/ml. **(B)** IgM-RF levels at different timepoints of exclusively the healthy participants who have an IgM-RF level above the cutoff (>3 AU/mL) already at day 0. The median IgM-RF level for each timepoint is indicated in red.


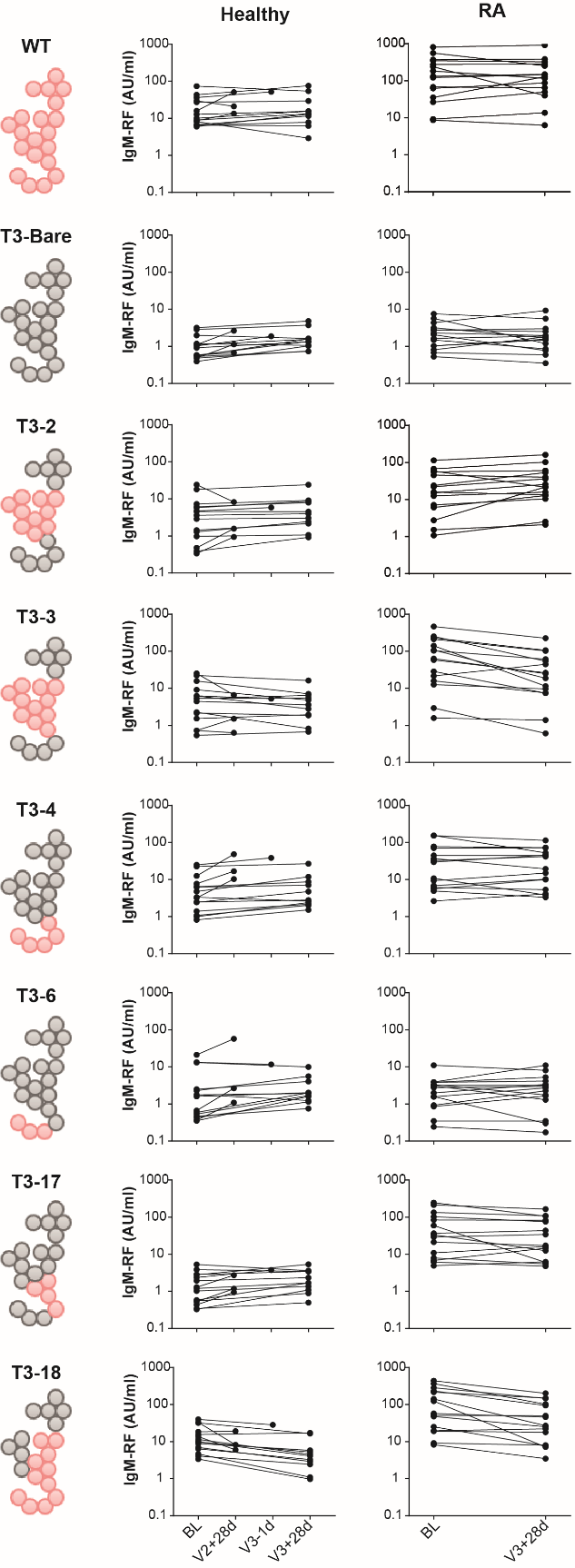


## Supplementary Figure 4. RF reactivity to different IgG targets.

IgM-RF reactivity to distinct binding epitopes of the IgG-Fc before and after the second or third vaccination in healthy participants. (left graph) or RA patients (right graph).

# References

7. Taha SI, Samaan SF, Ibrahim RA, El-Sehsah EM, Youssef MK. Post-COVID-19 arthritis: is it hyperinflammation or autoimmunity? Eur Cytokine Netw. 2021;32(4):83–8.

8. Xu C, Fan J, Luo Y, Zhao Z, Tang P, Yang G, et al. Prevalence and characteristics of rheumatoid-associated autoantibodies in patients with covid-19. J Inflamm Res. 2021;14:3123–8.

9. Schultheiß C, Willscher E, Paschold L, Gottschick C, Klee B, Henkes SS, et al. The IL-1β, IL-6, and TNF cytokine triad is associated with post-acute sequelae of COVID-19. Cell Reports Med. 2022;3(6).

10. Anaya JM, Monsalve DM, Rojas M, Rodríguez Y, Montoya-García N, Mancera-Navarro LM, et al. Latent rheumatic, thyroid and phospholipid autoimmunity in hospitalized patients with COVID-19. J Transl Autoimmun. 2021;4(February):1–7.

11. Lingel H, Meltendorf S, Billing U, Thurm C, Vogel K, Majer C, et al. Unique autoantibody prevalence in long-term recovered SARS-CoV-2-infected individuals. J Autoimmun. 2021;122(January):293.

23. Jarvis JN, Taylor H, Iobidze M, Dejonge J, Chang S, Cohen F. Rheumatoid Factor Expression and Complement Activation in Children Congenitally Infected with Human Immunodeficiency Virus. Clin Immunol Immunopathol. 1993;67(1):50–4.

24. Procaccia S, Lazzarin A, Colucci A, Gasparini A, Forcellini P, Lanzanova D, et al. IgM, IgG and IgA rheumatoid factors and circulating immune complexes in patients with AIDS and AIDS-related complex with serological abnormalities. Clin Exp Immunol. 1987;67:236–44.

25. Jackson S, Tarkowski A, Collins JE, Dawson LM, Schrohenloher RE, Kotler DP, et al. Occurrence of polymeric IgA1 rheumatoid factor in the acquired immune deficiency syndrome. J Clin Immunol. 1988;8(5):390–6.

26. Elkayam O, Segal R, Lidgi M, Caspi D. Positive anti-cyclic citrullinated proteins and rheumatoid factor during active lung tuberculosis. Ann Rheum Dis. 2006;65(8):1110–2.

27. Starshinova A, Malkova A, Zinchenko Y, Kudryavtsev I, Serebriakova M, Akisheva T, et al. Identification of autoimmune markers in pulmonary tuberculosis. Front Immunol. 2023;13(January):1–11.

28. Djavad N, Bas S, Shi X, Schwager J, Jeannet M, Vischer T, et al. Comparison of rheumatoid factors of rheumatoid arthritis patients, of individuals with mycobacterial infections and of normal controls: Evidence for maturation in the absence of an autoimmune response. Eur J Immunol. 1996;26(10):2480–6.

29. Sheagren JN, Tuazon CU, Griffin C, Padmore N. Rheumatoid factor in acute bacterial endocarditis. Arthritis Rheum. 1976;19(5):887–90.

30. Carson DA, Bayer AS, Eisenberg RA, Lawrance S, Theofilopoulos A. IgG rheumatoid factor in subacute bacterial endocarditis: relationship to IgM rheumatoid factor and circulating immune complexes. Clin Exp Immunol. 1978;31(1):100–3.

31. Gouriet F, Bothelo-Nevers E, Coulibaly B, Raoult D, Casalta JP. Evaluation of sedimentation rate, rheumatoid factor, C-reactive protein, and tumor necrosis factor for the diagnosis of infective endocarditis [1]. Clin Vaccine Immunol. 2006;13(2):301.

32. Cerny EH, Farshy CE, Hunter EF, Larsen SA. Rheumatoid factor in syphilis. J Clin Microbiol. 1985;22(1):89–94.

33. Meyer MP, Beatty DW. IgM rheumatoid factor in congenital syphilis: associations with clinical and laboratory findings. Clin Exp Immunol. 1991;86(1):43–8.

34. Tarkowski A, Czerkinsky C, Nilsson L-A. Simultaneous induction of rheumatoid factor-and antigen-specific antibody-secreting cells during the secondary immune response in man. Clin Exp Immunol. 1985;61:379–87.

35. Welch MJ, Fong S, Vaughan J, Carson D. Increased frequency of rheumatoid factor precursor B lymphocytes after immunization of normal adults with tetanus toxoid. Clin Exp Immunol. 1983;51(2):299–304.

47. Salonen EM, Vaheri A, Suni J, Wager O. Rheumatoid factor in acute viral infections: Interference with determination of IgM, IgG, and IgA antibodies in an enzyme immunoassay. J Infect Dis. 1980;142(2):250–5.

48. Zengin O, Yildiz H, Demir ZH, Dag MS, Aydinli M, Onat AM, et al. Rheumatoid factor and anti-cyclic citrullinated peptide (anti-CCP) antibodies with hepatitis B and hepatitis C infection: Review. Adv Clin Exp Med. 2017;26(6):987–90.

49. Philémon EA, Tume C, Okomo Assoumou MC, Tchuandom Bonsi S, Georges IM, Ouambo Fotso H, et al. A cross sectional study of the impact of human immunodeficiency virus, hepatitis b virus and hepatitis c virus on rheumatoid factor production. Arch Rheumatol. 2018;33(4):402–7.

50. Williams RC, Kunkel HG. Rheumatoid factor, complement, and conglutinin aberrations in patients with subacute bacterial endocarditis. J Clin Invest. 1962;41(3):666–75.

51. Asherson RA, Tikly M, Staub H, Wilmshurst PT, Coltart DJ, Khamashta M, et al. Infective endocarditis, rheumatoid factor, and anticardiolipin antibodies. Ann Rheum Dis. 1990;49:107–8.

52. Adebajo AO, Akinsola A, Maizels RM, Cawston TE, Hazleman BL. Rheumatoid factor and rheumatoid factor isotypes in loiasis with and without accompanying glomerulonephritis. Trans R Soc Trop Med Hyg. 1992;86(6):667–9.

53. Levinson AI, Tar L. In vitro IgM rheumatoid-factor production induced by tetanus toxoid. J Allergy Clin Immunol. 1988 Apr 1;81(4):730–6.
